# Cut wires: *The Electrophysiology of Regenerated Tissue*

**DOI:** 10.1186/s42234-021-00062-y

**Published:** 2021-02-23

**Authors:** Alexis L. Lowe, Nitish V. Thakor

**Affiliations:** The Neuroengineering Lab, Department of Biomedical Engineering, Johns Hopkins University, Baltimore, MD 21205 USA

**Keywords:** Nerve regeneration, Reconstructive surgery, Electrophysiology, Bioelectronics, Neural interfaces, Neuromuscular, Sensorimotor, Electromyography (EMG)

## Abstract

When nerves are damaged by trauma or disease, they are still capable of firing off electrical command signals that originate from the brain. Furthermore, those damaged nerves have an innate ability to partially regenerate, so they can heal from trauma and even reinnervate new muscle targets. For an amputee who has his/her damaged nerves surgically reconstructed, the electrical signals that are generated by the reinnervated muscle tissue can be sensed and interpreted with bioelectronics to control assistive devices or robotic prostheses. No two amputees will have identical physiologies because there are many surgical options for reconstructing residual limbs, which may in turn impact how well someone can interface with a robotic prosthesis later on. In this review, we aim to investigate what the literature has to say about different pathways for peripheral nerve regeneration and how each pathway can impact the neuromuscular tissue’s final electrophysiology. This information is important because it can guide us in planning the development of future bioelectronic devices, such as prosthetic limbs or neurostimulators. Future devices will primarily have to interface with tissue that has undergone some natural regeneration process, and so we have explored and reported here what is known about the bioelectrical features of neuromuscular tissue regeneration.

## Introduction: evolution of bioelectronics

There is a reason why many bachelor’s degree programs in neuroscience require that new trainees take a physics course on electricity and magnetism: understanding the nervous system requires a basic understanding of electronics. That is because nervous tissue performs most of its primary functions using electrical principles (Sterratt et al., 2011). Neurons, the cells responsible for generating, sending, and processing information, have evolved to use electrochemistry for sending bioelectrical signals in binary across the body by either being “off” or “on” (also called “spiking”). Neurons have detectable voltages across their cell membranes, which can change rapidly within milliseconds and be sent quickly over long distances (Nave, 2010). The largest and longest neurons are sensorimotor neurons that send bioelectrical signals between the spinal cord and the muscles of the extremities (Nicholson et al., 2000).

The relationship between the nervous system and electronics is what gives biomedical engineers hope that they can solve the problem of nerve damage. Nerve damage is a challenging fate to befall any vertebrate; whether through limb amputation, trauma, or disease, it is frustrating and disabling to have a part of the body no longer be under the control of the nervous system (Bailey et al., 2009). If an electrical cable is cut, thus interrupting signals to or from a device, the engineer’s solution is to repair the cable or give it a new adaptor. These options are possible because the exposed end of the cable is still conducting detectable electronic signals. A cut nerve is not so different, and it is this principle of trying to “plug into” a damaged nerve that helped to spawn the birth of bioelectronic medicine (BEM).

**Fig. 1:**
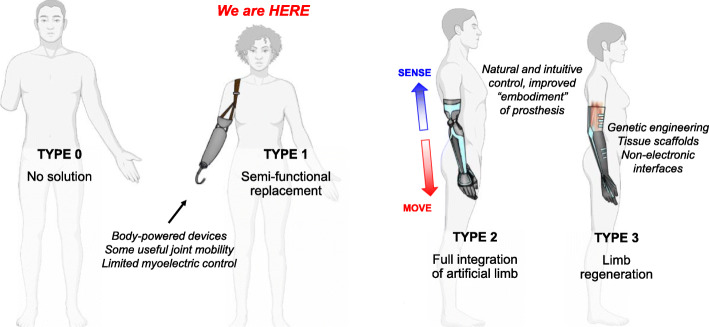
The Evolution of Bioelectronics. When it comes to the development of bioelectronic devices, the evolution of these devices’ use in medicine can be described with a scale based on Kardashev’s scale. A “Type 0″ bionic species has no ability to treat neuromuscular damage with electronics. “Type 1″ species are like modern humans. We can form stable mechanical interfaces with devices that partially replace the function of some nervous tissues. Passive bioelectronic devices like wearables, pacemakers, or cochlear implants have been adopted into mainstream medicine. Engineers are working to bring us closer to a “Type 2″ species, in which bioelectronic devices that would previously have been considered “active,” requiring conscious input from the user to function, can become fully integrated into the human nervous system for intuitive control. “Type 3″ species will have an even more harmonious integration of biology and technology, something that is likely beyond our predictive capabilities

BEM is a field that encompasses any use of electronic devices to interface with biological tissue for the purpose of treating disease (Olofsson & Tracey, 2017; Sanjuan-Alberte & Rawson, 2019). As BEM continues to evolve, so too will bionic humans, people that have had the functionality of a lost body part completely replaced by an electronic device. There is an obvious path for the ongoing evolution of bioelectronic devices and prosthetics, much like the Kardashev scale for the evolution of civilizations (Kardashev, 1985) (Fig. 1). A Type 0 bionic species has no solution for the loss of a body part. An example of this might be *Homo neanderthalensis*, which was a technologically intelligent species that simply did not have the generational knowledge required to make functional prostheses (Noonan, 2010). Type 1 bionic species, like modern *Homo sapiens*, have some basic assistive devices which can replace partial function of the body, but are limited in their total capabilities. For example, Type 1 prosthetic arms can perform some basic movements, grasps, and gestures, but they are still primarily tools requiring conscious control by the user (Smail et al., 2020). These devices cannot completely replace the high level of dexterity and sensitivity to touch that an intact human hand provides. Type 2 is the next stage of bionics and the goal for current research: seamless integration of artificial devices with the human body and nervous system (Yi et al., 2019). For example, Type 2 prosthetic arms would be used less like tools and more like natural extensions of the body. The control mechanism would be intuitive, not requiring any user training, and would provide dense sensory information that the user could subconsciously process. Type 3 species, practically unimaginable and far in the distant future, would be able to gain exponentially more functionality in terms of the energy invested into their prostheses (e.g., complete limb regeneration).

For the past few years, bioelectronics researchers have pointed out that the artificial robotic arms being used in modern prosthetics research can perform every mechanical function that a human arm can (Wormley, n.d.; Wodlinger et al., 2014; Moran et al., 2019). There are even electronic skins available that have been loaded with sensors for detecting textures, pressures, and temperature (García Núñez et al., 2019; Ma et al., 2019; Osborn et al., 2018; Sankar et al., 2020). So why, the question is often asked, is a perfectly natural-looking-and-feeling prosthetic arm a device exclusive to science fiction? It is likely because the jump from Type 1 to Type 2 is not limited by electronic technology, but is rather limited by a bioelectronic interface problem. When an ethernet cable is cut, the solution may be to simply attach a new connector, but the solution is not that simple when it comes to cut nerves. Nerve cells are regenerative and self-healing, which is excellent for the survival of organisms, but it is not favorable when making bioelectronic interfaces (Renz et al., 2018). Nervous tissue is highly sensitive to change, and any perturbation during its natural regeneration can affect the final biological outcomes (Schlosshauer et al., 2006). Not only will the nerve cells react to artificial interfaces, but also the lymphatic immune system will generate its own unique response to the foreign device as it impacts multiple body systems (Gulino et al., 2019; Hassler et al., 2011).

Damaged ethernet cables do not alter their own physical properties when a new connector is attached, but if they did, it would be logical to design new plugs based on how the cable changes after damage rather than how it behaves under normal conditions. If an engineer is planning to “plug into” an actively regenerating nerve within the human body, it will be necessary to know what the bioelectrical signals will look like and how they will change over time. The aim of this article is to review what the literature has to say on the subject of nerve regeneration in vivo and the electrophysiology of the reinnervated tissue. Much of BEM is focused on using bionics to treat nerve damage or malfunctions (Sanjuan-Alberte & Rawson, 2019). Therefore, it is important for biomedical engineers to understand the electrical considerations that must be made when interfacing with tissue after its recovery from major trauma.

## The electrophysiology of regenerated tissue

### Regenerating nervous system

Damaged nerves enter a regenerative state in response to trauma, altering their gene expression in an attempt to mate the damaged nerve ending with a new effector tissue, usually muscle (Johnson et al., 2005). The result of this regenerative process will largely depend on the composition of tissue that is surrounding the nerve’s stump (Dodla et al., 2019). If the nerve has no available effector targets nearby, the axons will begin to grow chaotically and can form a thick bulb of tissue called a neuroma (Balcin et al., 2009). Limb amputation is an example of nerve damage in which there is very little surrounding tissue left to act as an effector, and therefore over half of amputees experience neuroma pain (Hsu & Cohen, 2013). Fortunately, the intense local pain and some “phantom limb” pain that is caused by the neuroma can be prevented or reduced through surgical reconstruction (Poppler et al., 2018; Hart & Kung, 2020). There are two types of surgical interventions which have been most successful: nerve-nerve coaptation and autologous muscle grafts (Hart & Kung, 2020; Kang et al., 2019). Both methods have unique implications for what the final tissue electrophysiology will look like, but to understand these implications we must first explore the biology of this tissue.

**Fig. 2:**
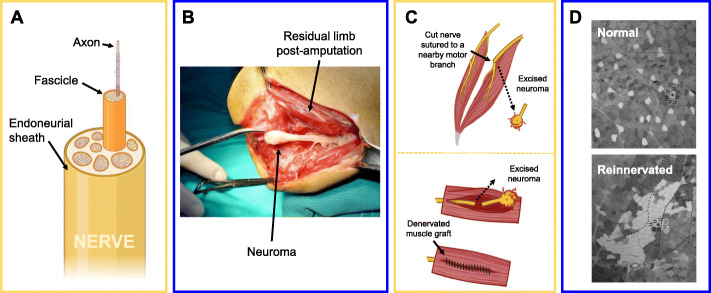
Regenerating Nervous System. **a** The cross-section of a nerve contains bundles of axonal projections traveling away from their cell bodies. The entire nerve is wrapped in an endothelial tissue referred to as the endoneurial sheath. Within the endoneurium, axons are grouped into fascicles that later branch out of the nerve. **b** A neuroma is a mix of fibroblasts, collagen, scar tissue, and neuron axons that have escaped from a cut nerve. This tissue is electroactive and known to cause severe pain in amputees (ehirlio lu et al., 2007). **c** To prevent neuromas from forming, a cut nerve ending can be sutured to the distal motor branch of a nearby nerve (a.k.a. nerve-nerve coaptation or TMR, shown on top). Another option for preventing pain is to wrap a denervated muscle graft around the cut nerve ending (shown on bottom). Both devascularized grafts (RPNIs) and vascularized grafts (VDMTs) have been demonstrated to prevent neuroma formation in humans. **d** Reinnervated muscle shows some very obvious and reproducible differences when compared to intact muscle. The most visually obvious change is the loss of a mosaic distribution among fibers from one MU. Instead of a mosaic, reinnervated muscle develops MUs in which all of the fibers in that unit are spatially clumped up together. Different shades of grey in this image of a muscle cross-section represent different fiber types, and darker fibers have higher oxidative capacity (less fatigability) (Brooke et al., 1971)

The biology is important because the biomolecular properties of axons and muscle fibers contribute to the voltages that are sensed by bioelectronic devices. Neuron cell bodies, which are the direct source of the voltages, are all located in or very near the spinal cord (Giuliodori & DiCarlo, 2004; Davis-Dusenbery et al., 2014). The axons of these cells, which conduct the voltages across the body, are all wrapped together like insulated wires inside of an endoneurial sheath (Fig. 2a). The axons are further bundled into fascicles, which contain a mix of sensory and motor neurons (Stewart, 2003). When a nerve is cut in the periphery, the part of the nerve that is separated from the cell body immediately begins to swell and degrade (a.k.a. Wallerian degeneration) (Johnson et al., 2005). If the cut is so complete that all of the axons and the endoneurial tube have been severed, the axon endings will no longer have a reliable guide to follow back towards their effector. Evidence supports the theory that a neuroma forms once the axons sprouting from the end of the tube run into scar tissue that is forming within other portions of the wound site (Foltan et al., 2008; Sisken et al., 1989). The fibrous scar tissue contracts around the axons, and the result is a thick, white bulb of cells and collagen at the nerve ending which is incredibly hypersensitive and painful, with or without being physically touched (ehirlio lu et al., 2007) (Fig. 2b). The best way to prevent neuromas is to place a new effector tissue at the nerve stump to promote reconnections and provide neurotrophic support to the nerve (Dellon & Aszmann, 2020).

The historical solution for preventing neuroma formation after amputation was to bury the free nerve ending into a nearby muscle, keeping it safe from scar tissue encapsulation (Foltan et al., 2008). The muscle in this case is already innervated, so it does not make a bioelectrical connection with the nerve. Neuromas still occur in more than 50% of these bury-in-muscle surgical cases (Poppler et al., 2018). Forming a stable, bioelectrical connection between the nerve and muscle is the best known solution for preventing neuroma pain, and it is also useful for future prosthesis wear (discussed more in the next section) (Hart & Kung, 2020). Targeted muscle reinnervation (TMR) is a surgical technique in which the cut nerve is sutured to the distal end of a nearby motor nerve through what is called “nerve-nerve coaptation.” (Fig. 2c). TMR is useful because it does not disturb the vascularization or neuromuscular junctions (NMJs) of the target muscle. The downside is that an upper-limb sensorimotor nerve is often quite thick, and the donor nerve stump can be small in comparison. There have been concerns that this size mismatch leads to some axons degrading, some of them even escaping and forming nearby neuromas after surgery (Dellon & Aszmann, 2020).

The other surgical option for neuroma prevention is to denervate a piece of muscle and then wrap it around the cut nerve ending directly (Fig. 2c). If the muscle is devascularized (a free-floating graft), then it is considered a regenerative peripheral nerve interface (RPNI), popularized by the Cederna group at the University of Michigan (Kung et al., 2014; Kubiak et al., 2018; Ursu et al., 2016). If the muscle is still leashed by its native vasculature, it is considered a vascularized denervated muscle target (VDMT), developed by the Tuffaha group at Johns Hopkins University (Tuffaha et al., 2020). It is not yet known which of these surgical interventions provides the best pain relief results or the best prosthetic control, but it is possible to make predictions about the cellular physiology based on our knowledge of how muscle cells change in response to reinnervation.

Nerves and muscles are bioelectrically connected through NMJs. The NMJ is a synaptic connection between the end of a motor neuron and a collection of muscle fibers (Sanes & Lichtman, 1999). At the interface of the two cells, the neuron releases acetylcholine into the synaptic space whenever it fires. The acetylcholine binds to receptors on the muscle fiber, which then opens up ion channels that cause the muscle fiber to quickly change its membrane voltage and begin contracting (Burden, 2011; Hopkins, 2006). When a muscle is denervated, these ion channels can become hypersensitive and somewhat “leaky,” which is thought to be the reason why denervated muscle is prone to random spasms, also referred to clinically as fasciculations (Carlson, 1981; Reimers et al., 1996).

A motor unit (MU) is defined as all the muscle fibers that are connected to a single motor neuron. It is the firing rate of that neuron which determines what the muscle fiber type will be, so all the fibers in one MU are of the same type (Farina et al., 2016). Muscle fibers can be one of three types: slow oxidative (type I), fast oxidative (type IIA), or fast glycolytic (type IIB) (Brooke et al., 1971). “Fast” or “slow” refers to the fiber’s speed of contraction. Oxidative fibers produce ATP (chemical energy) through aerobic respiration, so they have more ATP and are slow to become fatigued. Glycolytic fibers primarily use glycolysis (an anaerobic process) and can run out of energy quickly. Normally, the fibers of a MU are spread out across the muscle, forming a heterogeneous mosaic (Brooke et al., 1971). When a muscle is denervated, the immediate physical change is that the muscle fibers atrophy (Carlson, 1981). Connecting the muscle with a new nerve can help reverse some of this degeneration, but it also causes the fiber types to change dramatically. First, the motor units, rather than being intermingled, become clumpy, which is sometimes referred to as “fiber type grouping” (Gordon & de Zepetnek, 2016) (Fig. 2d). Next, the fibers change their type based on the nerve’s activity. For example, if a fast-twitch muscle is reinnervated by a nerve that came from a slow-twitch muscle, then the reinnervated muscle will develop a larger percentage of type I fibers (Brooke et al., 1971).

All of these cellular changes have a direct impact on the electromyography (EMG) signals that the muscle produces. Muscle cells can only maintain a voltage across their membranes so long as they have sufficient ATP to fuel the proteins responsible for maintaining the fiber’s length and electrochemical gradients. As far as muscle function is concerned, it has been shown that both free muscle grafts and muscles attached to the skeleton are capable of physically contracting after reinnervation (Vu et al., 2020; Stubblefield et al., 2009; Hu et al., 2020). The peripheral nervous system (PNS) behaves like an adaptive circuit, and to plug into that circuit, it is relevant to piece apart how each component, in series or in parallel, will affect the electrical output.

### Nerves and wires

When analyzing a circuit, a good place to start is with the signal source. In this case, that source is the cut sensorimotor nerve. Some readers might object to that statement because when it comes to moving our bodies, increasing our heart rates, and distinguishing sensory inputs, the real signal source can be traced all the way back to the central nervous system (CNS): the cerebrum, cerebellum, brainstem, and spinal cord. This is true, and many detailed investigations have been launched into interfacing with the CNS for the purpose of restoring lost function to people who are dealing with quadriplegia, tetraplegia, locked-in syndrome from ALS, or other neurodegenerative disorders (Andersen et al., 2004; Lebedev et al., 2011; Vidal et al., 2016; Jackson & Zimmermann, 2012). In this review, we will not be discussing bioelectronic interfaces with the CNS because the CNS is not capable of the same level of regeneration that is observed in the PNS. Neurons of the CNS do not have the same protein expression changes after trauma that are seen in peripheral nerves, and the CNS neurons also do not have the same kind of support cells in the immediate environment to help guide regeneration (Fenrich & Gordon, 2004). Now that we have narrowed down the subject of our exploration, we can build our discussion of the electrical characteristics that are known about the primary signal source for most bioelectronic interfaces: peripheral nerves.

The diversity in the design of peripheral nerve interfaces has blossomed over the past decade (recently reviewed by Cho and colleagues), but the majority of those interfaces are stimulatory (Cho et al., 2020). By comparison, very few *recording* devices have been utilized in vivo, limiting the depth of our knowledge regarding normal electroneurography (ENG) signals in mammals. There are several factors that introduce bottlenecks to the process of in vivo ENG recording. First, nerve voltages are small, on the order of microvolts (10^− 6^ V), and muscle contractions cause detectable voltages on the order of millivolts (10^− 3^ V). Muscles, therefore, produce a lot of noise during in vivo ENG recordings (Loeb & Peck, 1996). Second, nerves are physically small, squishy (*compliant*, as an engineer might say), and they can be very sensitive when they are put into contact with stiff electrodes (Wellman et al., 2018). Any mechanical mismatch can lead to a breakdown of the interface, making a chronically-implanted electrode useless over time (Lacour et al., 2010; Spearman et al., 2020). Third, the structure of a nerve ensures that many of the axons carrying vital information are in the very core of the nerve, which makes it difficult to selectively record from those cells without penetrating the tissue and risking cell damage.

Most peripheral nerve electrodes aim to record from healthy, intact portions of the tissue because that healthy portion contains axons from all the neurons necessary to drive a muscle. Additionally, such electrodes can be tested in vivo simply by anesthetizing an animal subject, performing a small exposure surgery, and then testing the electrode on a healthy nerve for a few hours, possibly even implanting the electrodes for a few weeks (Elyahoodayan et al., 2020). Developing electrodes that specifically interface with *regenerated tissue* requires months of time invested into painstakingly recording from a damaged nerve as it recovers millimeter by millimeter (Johnson et al., 2005). Larson and Meng recently published a meticulous review that dives deeply into the design considerations for electrically interfacing with healthy nerves (Larson & Meng, 2020). Our review, to iterate, is primarily concerned with the electrophysiology of regenerated tissue, so we will only review the details of electrodes designed to interface with regenerated neurons. Currently only two electrodes truly fit that description: sieve electrodes and the Substrate Targeted Electrode Reinnervation (STEER) device (Russell et al., 2019; Blasiak et al., 2019).

**Fig. 3:**
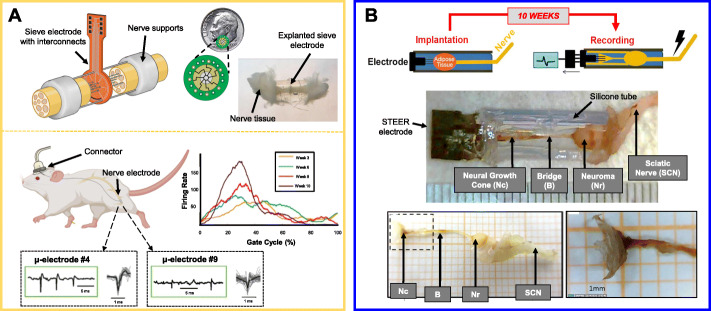
Regenerative Nerve Interfaces. **a** Sieve electrodes are structured as porous disks, allowing a cut nerve ending to travel through the channels with electrode contacts after nerve-nerve coaptation surgery. Single unit action potentials have been evaluated during sciatic nerve regeneration in rats, showing that neuron firing rates increased over the course of 10 weeks (Musick et al., 2015). At the top is an illustration of a sieve electrode with interconnects for ENG recording as well as an image of an explanted that sieve electrode that was previously implanted in an animal (Freudenrich, 2007). At the bottom is a demonstration of how longitudinal ENG recordings can be taken from rodents via a head-mounted connector while walking. **b** The STEER electrode is a neuroma interface, designed to limit neuroma growth to within a 150 mm long silicone chamber (top) (Lahiri et al., 2016). Ten weeks after the sciatic nerve of 5 rats was cut and implanted into the STEER electrode, a neuroma had formed (middle). The neuroma was only a few millimeters long, and some unexpected neuro-fibrous tissue structures were found to grow within the rest of the silicone tube (middle and bottom). At the very end of the chamber, a neural growth cone formed over the electrode wires, and the tissue produced electrically-evoked compound APs of a few hundred microvolts. Future STEER electrodes can have more detail for the purpose of guiding the neural growth cone towards making more contacts with the electrode sensor (Blasiak et al., 2019)

Sieve electrodes were designed to address the spatial recording problem that was previously mentioned. These electrodes are round and contain many holes, so when a nerve is cut cleanly, both the proximal and distal ends of the nerve can be sutured to either side of the electrode (Jinwoo et al., 2016; MacEwan et al., 2016). As the nerve regenerates, axons grow through the electrode and make contact with the sensors lining the inside of each channel (Freudenrich, 2007; Panetsos et al., 2008) (Fig. 3a). This kind of electrode should detect ENG signals from neurons deep inside the core of the nerve just as easily as it can detect signals from the outer neurons. A generic sieve electrode has rows of round channels, but many different designs have been considered for these regenerative interfaces (Delgado-Martínez et al., 2017; Clements et al., 2013; Garde et al., 2009). There are even bifurcating designs – elongated tubes with channels that split into smaller channels for higher spatial resolution – that have shown to be viable in vivo (Stoyanova et al., 2013; Wieringa et al., 2010). (For the sake of simplicity, though, we will use “sieve electrodes” as an umbrella term for all devices that rely on nerve-nerve coaptation surgery.)

In 2015, Srinivasin and colleagues cut the sciatic nerve of 5 rats and implanted a silicone-based sieve electrode between the two nerve endings during nerve-nerve coaptation surgery. The electrode was left completely implanted and undisturbed for 5 months (Srinivasan et al., 2015). After the 5 months of regeneration, the animals were anesthetized, the implant was accessed surgically, and then the electrodes were plugged into an external recording device. Out of the 40 electrode channels tested (8 per device), 9 channel leads were broken, and 8 channels could reliably be used to detect single-cell action potentials (APs), which is the more technical term for neuronal “spikes.” The authors could identify unique waveforms of the different APs, which had amplitudes of 40–80 μV. The firing of these cells could be stimulated by brushing or moving the animal’s foot, which indicates that they were making some electrical contact with sensory neurons. In that same year, Musick and colleagues also implanted a silicone-based sieve electrode into the sciatic nerve of rodents (Musick et al., 2015). They implanted their electrodes into 4 Lewis rats and ran the electrode leads subcutaneously towards the animal’s head. Every week for 7 weeks post-recovery, the ENG signals of each animal were recorded via a permanent, head-mounted connector as the animal consciously walked down a runway. Out of 40 electrode channels tested (10 per device), all 40 channels could reliably detect single-cell APs with an amplitude of 20–60 μV at 6 weeks post-operation. From week to week, as the nerves regenerated through the electrode, the authors observed a gradual increase in the firing rates of cells (measured in APs per second). The firing rates seemed to grow in correlation with a specific part of the animal’s gait cycle while it was walking. This is the only example we could find of a dataset that includes longitudinal data recorded from a multichannel sieve electrode. This report is in fact a rare observation of ENG signals from a regenerating nerve in vivo. This rarity is to be expected considering all of those previously-mentioned bottlenecks as well as the fact that successfully keeping rats with chronically-accessible implants is not an easy task (Clements et al., 2013).

The STEER electrode, first demonstrated in 2016, is also limited by a lack of available longitudinal data, but it is unique enough to be worth mentioning in this review. The primary reason is that the STEER electrode is less of a neural interface and more of a *neuroma* interface. The STEER electrode is simple: it is a silicone tube with a tungsten microwire electrode array inside one end and an opening at the other end for a cut peripheral nerve to enter (Fig. 3b) (Blasiak et al., 2019; Lahiri et al., 2016). During implantation, a piece of ischemic adipose tissue from the animal is placed into the tube between the electrode and the nerve ending. This fatty tissue is present to promote fibrosis, ensuring that the cut nerve ending will form a neuroma inside of the tube. The purpose for intentionally promoting a neuroma was to see if the fibro-axonal-collagenous bulb, which is very stiff compared to normal nerve tissue, would form a more stable physical connection with the artificial interface. Previous work had shown that neuromas still contain functional axons despite being much stiffer than healthy tissue (Amir & Devor, 1993; Macias et al., 1998; Maki et al., 2005). Lahiri and colleagues implanted STEER electrodes into the hindlimbs of 5 female rats for 10 weeks. After explantation it was obvious that the neuroma did form, but there was also something totally unexpected. Some of the axons had continued to project out of the neuroma bulb to form a bridge towards the microwire electrode, where a cone-like structure consisting of axons, fibroblasts, and collagen had formed. During explantation, the authors stimulated the sciatic nerve upstream of the device, and recorded voltages from the fibro-neuronal structure. The electrodes recorded compound APs, which are the result of multiple neurons firing synchronously, with amplitudes ranging from 10 to 500 μV. Different from normal nerve tissue, the neural growth cone was found to have lower conduction velocities, likely due to the incorporation of fibroblasts into the structure.

Regenerative nerve interfaces are rare, and are not the most practical when it comes to bioelectronic recording. It is still a technological challenge to longitudinally record from an implanted interface (Grill et al., 2009). This is because keeping animals with head-mounted connectors is a difficult endeavor, both for the biomedical engineer and for the animals who must tolerate a chronically-open wound in order to connect the implanted device with an external recording apparatus. Whether through interference from the animal or from the physical stress of being implanted in a moving tissue, prototype device failures in vivo are common (Shafer et al., 2019; Kuliasha et al., 2019). Robust longitudinal data will become more accessible whenever wireless recording technology can be easily obtained in the lab (Cho et al., 2020). The ideal wireless recording system does not yet exist partially because the process of transmitting high-frequency ENG data through tissue, through the air, and into an external device is energy-expensive (Teshome et al., 2019).

Unlike recording devices, neurostimulators can operate autonomously and wirelessly within the body for over 10 years using similar batteries to pacemakers (Edwards et al., 2017; Sette et al., 2019). Chronically-implanted neurostimulators have been successful in humans and are even commercially available for clinical use (e.g., cochlear implants, vagus nerve stimulators, deep brain stimulators, etc.) (Marin et al., 2018; Kassiri et al., 2017). Somatosensory neurostimulation has been tested on human amputees, both through implantable and dermal electrodes (Dimante et al., 2020). Much of this work has demonstrated that somatosensory feedback can improve the ease-of-use and the embodiment of prosthetic limbs by their users (Antfolk et al., 2013; Petrini et al., 2019). These nerve stimulation devices still have a long way to go before humans can be considered to have achieved Type 2 bioelectronic sensory feedback, the kind of feedback that is sensitive, fast, and accurate enough to fully replace one’s original sense of touch. Several ways in which direct neurostimulation can be improved are detailed more in the Discussion section of this article, but briefly, those areas of improvement tend to fall under one of two categories: device fabrication or sensory information encoding (HajjHassan et al., 2008; Iskarous & Thakor, 2019). On the reverse side, in order to improve how bioelectrical signals are *extracted* from the nervous system (rather than *administered* to it), the most popular and promising way to begin is by first “plugging” the motor nerve of interest into its natural amplifier: muscle.

### Muscles and amplifiers

Earlier we discussed how surgically forming a bioelectrical connection between a cut nerve and a new muscle target will help to prevent a neuroma from forming. This is true, but the original purpose of these reinnervation surgeries was actually to generate bioelectrical signals that are easier to detect for controlling a prosthetic limb. The voltage detected from muscle tissue is orders of magnitude larger than the voltage detected from nerves, and because the two signals are directly correlated with each other, muscles are often referred to as “bio-amplifiers” (Vu et al., 2020).

**Fig. 4:**
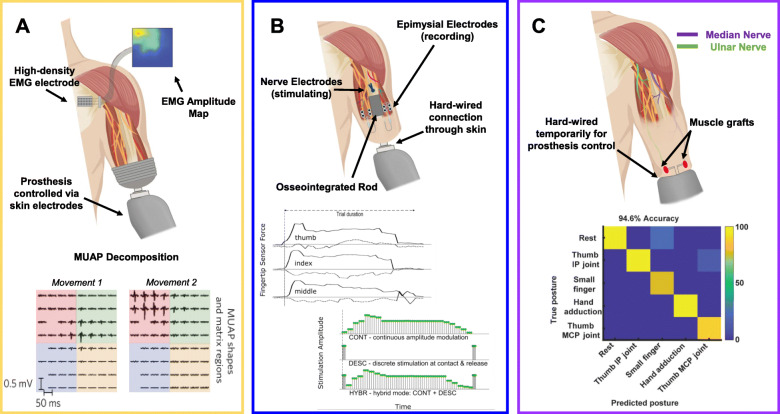
Reinnervated Muscle Interfaces. **a** The most common signal used for controlling myoelectric prostheses is sEMG. These voltages are detected from the skin through removable electrode contacts (top). The most accurate signal interpretation is through motor unit decomposition. An sEMG signal is broken down by an algorithm into the individual firing rates of unique motor units (bottom). This algorithm requires high-density surface electrode arrays (Farina et al., 2017). **b** Implanted electrodes can be used to record eEMG signals for prosthesis control. Currently, the best method for doing this in human subjects is to connect the electrodes with an osseointegrated implant that interfaces with the external prosthesis, a.k.a. the e-OPRA (top). These implants require that there be a permanent opening in the skin. The e-OPRA presents an opportunity for developing stable bi-directional interfaces. eEMG signals can control the robotics, and nerve cuff electrodes implanted in the residual limb can be used to provide stimulation based on signals from artificial touch sensors that are within the prosthesis (bottom) (Mastinu et al., 2020). **c** RPNI muscle grafts have been shown to be useful for prosthesis control. The only published human trials so far recorded iEMG signals using temporary bipolar electrodes that were implanted percutaneously in the RPNI muscles of 2 upper-limb amputees (top). Simple amplitude-based classification algorithms could be used to reliably operate a prosthetic hand through a series of complex gestures by using the iEMG signals recorded from multiple RPNIs (bottom) (Vu et al., 2020)

EMG signals can be reliably detected even at the level of the skin, which is how modern myoelectric prostheses work (Markovic et al., 2020). An amputee will wear an electrode array on the surface of his/her residual limb, and the surface EMG (sEMG) signals will be recorded and fed into a classification algorithm (Fig. 4a, top). Certain signal patterns from different electrodes will initialize the robotic prosthesis to change its configuration, such as opening and closing a robotic hand. This technique works for recording from residual muscles within the stump or from surgically reinnervated muscle. When it comes to recording from reinnervated muscle, the electrophysiology of TMR has probably been studied the most in humans.

In 2016, Kapelner and colleagues (in a multi-national effort) published the results of their experiments characterizing the motor unit properties of reinnervated muscle after TMR surgery in humans (Kapelner et al., 2016). They recorded sEMG signals from 5 subjects with TMR and 9 control subjects by spreading electrode contacts over a large area of the pectoral muscles, which had been reinnervated in the TMR subjects (Farina et al., 2014). The raw sEMG signals do not look anything like the high-frequency spiking of motor neurons because skin and fat cells between the muscle and the electrode distort the signals like low-pass filters (De Luca et al., 2006). To find the firing rates of individual motor units, the authors needed high-density surface electrode arrays (8 × 8 contact grid with 10 mm spacing) and a decomposition algorithm (e.g., Convolutional Kernel Compensation) (Farina et al., 2014). The algorithm’s job is to “decompose” the raw sEMG signal to identify motor unit spike waveforms and their firing rates (Fig. 4a, bottom). Decomposing an sEMG signal is like listening to the recording of a symphony orchestra and then determining the exact musical notes being played by each instrument.

Once the raw EMG signal is decomposed, the firing rate of the different motor units can be associated with the voltages sensed by the surface electrodes to estimate where each motor unit is located. The authors found that many of the motor unit action potentials (MUAPs) were comparable between the TMR amputees and the able-bodied control groups, including similar firing rates and amplitudes around 150–200 μV. The key differences were that MUAPs from the TMR group were about 33% shorter, and the mean normalized area of motor unit territories was less than half that of the able-bodied group. Spatially, the motor units of TMR muscles were smaller and more likely to be overlapping with each other rather than being spread out with unique spatial territories.

Many of the authors from this TMR study (including Kapelner) were involved in follow-up studies, such as Farina and colleague’s *Nature BME* paper the following year (Farina et al., 2017)*.* The authors demonstrated how motor unit decomposition of sEMG from TMR amputees can be used for accurate control of a prosthetic arm. The MUAP estimations led to better signal classification than using just the electrode voltage amplitudes, which makes sense given that multiple MUs can be detected within the same electrode territory. At least 10 distinct MUs could be detected from all the TMR subjects, and there did not appear to be a direct correlation between the number of detected MUs and the classification accuracy. Other reports have also shown that MUAP decomposition of reinnervated muscle signals can be used for accurate signal classification and improved force estimation for prosthesis control (Sartori et al., 2019; Zheng & Hu, 2019). Most of these results, including those from Farina et al., were obtained during off-line classification. This means that the sEMG signals were recorded from a human subject, and then they were analyzed and classified sometime later on a computer. Amplitude-based EMG classification appears to be less accurate than MUAP decomposition, but at least it can be used to operate a prosthetic limb in real-time. The primary reason that the amplitude of an sEMG signal is the most common signal feature used by classification algorithms is that it is an easy feature to calculate. MUAP decomposition has a computational lag time, and so far, the most reliable way to reduce the lag time is to eliminate the computation all together. Directly sensing MUAPs instead of having to estimate them from sEMG signals requires that the EMG electrode interface be very small, sensitive, and very close to the muscle fibers – i.e., it requires implantable microelectrodes.

There are a few people today who have had amputations above the elbow, later had TMR surgery within the residual limb, and also had EMG electrodes implanted at the surface (also known as the *epimysium*) of the reinnervated muscle (Ortiz-Catalan et al., 2020) (Fig. 4b, top). These folks also have an osseointegrated rod implanted at the end of their residual limbs. This implant, more specifically known as the e-OPRA implant, crosses through the skin to offer a wired connection straight to the reinnervated muscle (Ortiz-Catalan et al., 2020; van der Kaaden, 2018). Myoelectric prosthetic arms can be directly plugged into this metal rod, so the epimysial EMG (eEMG) signals travel directly into a control processor that is outside the body. Moreover, the interface can be made bidirectional by adding stimulatory cuff electrodes around the sensorimotor nerve within the residual limb. The e-OPRA has shown promising clinical outcomes in its ability to operate a robotic hand for opening, closing, and pinching motions as well as to provide some level of sensory feedback to indicate the amount of force being applied by the prosthesis during grasping motions (Mastinu et al., 2019; Mastinu et al., 2020) (Fig. 4b, bottom).

The electrodes of the e-OPRA are single-channel bipolar electrodes, usually with one electrode attached to each muscle. One study by Mastinu and colleagues followed 2 human amputees who had each received their implants and TMR in a single surgery (Mastinu et al., 2018). Each subject had 4 electrodes, 1 electrode on each of the 2 different reinnervated muscles (radial and ulnar nerves), and 1 electrode each on the intact heads of the bicep and triceps. Over the course of 48 weeks post-operation, the eEMG signal-to-noise ratio (SNR, a metric for signal strength) of the two reinnervated muscles in both subjects increased from 0 dB to 20–30 dB, while the SNR was constant for the bicep and triceps muscles. Due to there being only one channel recorded per reinnervated muscle, there is no spatial information available regarding the electrophysiology of these subjects.

While there is not yet any spatial EMG data from implanted human subjects, there have been multichannel electrodes implanted into rodents after TMR surgery (Bergmeister et al., 2019; Muceli et al., 2018). These studies found that in a forelimb TMR model, the reinnervation of the bicep muscles by the ulnar nerve resulted in a “hyper-innervation.” More motor units could be detected after reinnervation compared to the control bicep muscle, both histologically and through electrophysiology. Spatially, the motor units were found to cover overlapping territories, with the strongest signals coming from the regions immediately next to the nerve-muscle interface. The NMJs were clumped around this area, but the reinnervated MUAPs were not found to be different from control MUAPs in amplitude or conduction velocity.

TMR has been well studied in human amputees because it has been a reliable form of amplifying the bioelectrical signals of the nervous system. Despite the technique’s popularity, TMR is not an easy surgery to perform because it requires the suturing of two nerve endings with very different diameters (Garg et al., 2018). This is certainly a situation that could benefit from implanting some kind of regenerative interface device, similar to a sieve electrode, that could guide the nerves toward their new effector tissue. Rather than focusing on this nerve-to-nerve connection, though, other surgeons have found success with the autologous muscle graft solution. Rather than trying to solder two wire endings together, turn the cut wire ending into a new plug. That is more or less the goal of the RPNI and the VDMT muscle grafts (Tuffaha et al., 2020; Urbanchek et al., 2016).

In 2016, the Cederna group at the University of Michigan reported the effects of RPNI surgery on neuroma treatment in adult amputees (Woo et al., 2016). The majority of subjects (77%) reported a decrease in their neuroma pain after the surgery. Four years later, the Cederna group in collaboration with Chestek’s engineering laboratory demonstrated that these small muscle grafts, years after the original RPNI surgery, produced EMG signals reliable enough to accurately control a robotic hand prosthesis (Vu et al., 2020). Both of the volunteer subjects had amputations at the wrist performed many years prior to receiving RPNI surgery. One subject had 2 RPNIs that were used for recording, and the other subject had 3. Both participants had one bipolar electrode temporarily implanted into each RPNI, and the intramuscular EMG (iEMG) signals were used to control the prosthetic hand (Fig. 4c). What was remarkable about this study was the accuracy with which the subjects could control a highly-dexterous thumb prosthesis and switch between hand positions to perform motor tasks in real-time. The amplitudes of iEMG signals were comparable to (if not a little larger than) the eEMG signals detected by e-OPRA devices. The RPNI method is promising, and it has been performed in several animal models including monkeys (Irwin et al., 2016; Sando et al., 2016). Those animal studies do not yet include comprehensive longitudinal recordings nor do they include multichannel recordings from the same piece of muscle. The same limitation is true for VDMT research, which has only just begun to include electrophysiological data. The results of future studies will be important to watch, as it has not yet been investigated whether muscle vascularization has an effect on the oxidative capacity or electrophysiology of reinnervated muscle fibers.

Reinnervated muscle turns out to be a very useful clinical tool and a strong candidate for interfacing with bioelectronic devices. The tissue’s natural amplification capabilities are worth investigating in a larger variety of contexts, especially with the rise of both cellular and acellular device components being used in bioelectronics (Weigel et al., 2018; Li et al., 2020). New biomaterials will continue to play a big role in the future development of EMG recording devices. Not only is there an interest in how EMG signals change over time, there is also an interest in how implantation changes the electrode’s sensing capabilities over time, which is a major challenge for material scientists. The long-term goal for sensorimotor neural interfaces is to understand the biological tissue well enough that devices can be designed to “plug into” the muscle with the same sensitivity and ease that headphones can be plugged into a jack. In the same vein, the future advancements of electronic devices in parallel with our understanding of bioelectric processes will likely lead to completely wireless signal detection solutions (just like what happened to the headphone industry).

## Discussion: from type 1 to type 2

Progress from both within and outside of academia has injected exciting energy into the field of bioelectronic medicine, and it seems more likely all the time that humans will make the jump from a Type 1 to a Type 2 bionic species within this century. It is possible that this jump will be achieved by having individual research teams systematically address the problems plaguing current Type 1 bioelectronic devices, producing the incremental improvements that we need slowly over time. Another possibility is that some evolutionary accident or serendipitous synergy among groups of researchers will produce an unexpected BEM solution that completely shifts paradigms. We would like to conclude this review article with a frank discussion of the problems that still need to be addressed for the development of future bioelectronics. Some of our proposed solutions will be obvious to other engineers within this field, but we hope to also stay edgy by introducing a few less-traditional biomedical possibilities that have become more feasible in recent years.

### Physical Interface problem

It has been certainly demonstrated by the articles cited within this review that modern bioelectronics suffer from a physical interface problem. Addressing this problem, making the perfect neural connector to plug into, can partially be addressed by improvements in microfabrication (HajjHassan et al., 2008; Sung et al., 2020; Zhu et al., 2014). The ideal electrode interface would be able to very selectively record from and/or stimulate unique neurons within a large pool of cells, which means that individual electrode leads should be less than a few micrometers thick. Electrode impedance will have to be low, which becomes increasingly challenging at smaller scales, and the SNR during recording must be high (Ward et al., 2009). The materials used would likely be very compliant, and the whole device must be encapsulated such that it both protects the internal electronics and does not stimulate a fibrotic immune response from the host (Ahn et al., 2019). Fixing the bioelectronic interface problem will also require better wireless electronics. The ideal bioelectronic interface could be implanted and then essentially left alone, much like a modern pacemaker that has well-enclosed electronics and very simple data transmission systems (French, 2020). Implantable ENG and EMG recording electrodes will be much more useful for longitudinally investigating the in vivo electrophysiology of regenerating tissues once the electrodes can be incorporated into a fully-encapsulated system that allows engineers access to high-quality, wireless signal data.

This list of device requirements that we have laid out can certainly be tackled in a systematic way by a skilled team of engineers using the scientific method; it is just a matter of time. It does not seem right to imply, though, that making the perfect physical neural interface will be a cure-all solution for BEM. If we break down the jump from Type 1 to Type 2 in terms of the *functional* improvements that must be made to bioelectronic devices, then it becomes more obvious how alternative methods and fields outside of microelectronic fabrication could be used to make evolutionary leaps. The primary functional changes we want to see in our current devices are (1) increased precision in the control of artificial effectors and (2) more naturalistic sensory feedback provided to the user.

### Inputs and outputs

In order to engineer a system that helps people like amputees to regain limb control and dexterity, the system input is usually some bioelectronic signal and the output is the movement of a robotic limb. Modern prosthesis control using bioelectronic interfaces would not be possible without the computer programming that is required to correlate certain bioelectric signals with electronic commands. The MUAP decomposition method of transforming a sEMG signal into a set of MUAP spike trains is a good example of how algorithms can make up for some of the shortcomings at the bioelectronic interface. One specific shortcoming of dermal interfaces (besides the EMG signal filtering) is the physical instability. The electrodes have a tendency to slip and shift during normal use and can be affected by environmental factors like sweat (Hargrove et al., 2006). Betthauser and colleagues recently demonstrated that applying basic modeling principles to their EMG classification algorithm allowed them to compensate for errors like those that are caused by electrodes shifting (Betthauser et al., 2019). The implementation of similar principles, as well as the development of better machine-learning prediction algorithms, could make it possible to have future devices that can precisely predict the proper orientation and applied force for an artificial limb using non-invasively-acquired signals. The technological challenge here involves writing all of the necessary computations and then setting them up to run on a lightweight, low-power computer inside of a mobile prosthesis in real-time – which sounds like another list of product requirements to be systematically tackled by future engineers.

Increased understanding of MUAP decomposition as a classification method has led to an increase in high-density surface recordings being taken from muscles (Hassan et al., 2019; Chen et al., 2020a; Chen et al., 2020b; Dai & Hu, 2019). In this review we outlined how tissue regeneration can impact the spatial features of the EMG signals detected by these multichannel arrays. It has yet to be examined whether or not the spatial layout of EMGs can be manipulated during the process of reinnervation with either electrical or pharmaceutical interventions. Experiments have been performed in the past to examine what happens to some temporal EMG features and biological tissue properties when reinnervated muscle is electrically stimulated during regeneration, but spatial signal data is comparatively absent (Zealear et al., 2002; Gordon et al., 2008; Asensio-Pinilla et al., 2009). There have also been some interesting investigations into whether or not sensory and motor neurons can be cleanly separated via chemical cues that are incorporated into regenerative neural interface devices, increasing the selectivity for recording versus stimulation (Lotfi et al., 2011; Kim & Romero-Ortega, 2012).

The scarcity of signal data from regenerated neuromuscular tissue (particularly *standardized* data) is the main obstacle preventing the development of comprehensive bioelectrical models. A good bioelectrical model would be able to predict cellular and tissue voltages based on biological features such as muscle fiber types, muscle size, nerve diameter, muscle blood perfusion, etc. Essentially, an accurate bioelectrical model would make our entire review article redundant. Being able to simulate biological environments and predict the electrical outcomes would be the first big step towards designing bioelectrical devices completely in silico, without ever having to make a physical prototype. For example, an accurate physical model of an amputee’s residual anatomy in this simulation, likely acquired through imaging methods such as MRI, could be analyzed to design a custom bioelectronic interface. The interface device could be tailored such that it uses data from as few channels as possible in order to conserve energy for data transmission. For this kind of modeling to play a part in the journey from Type 1 to Type 2 bioelectronics, high-quality and easily-accessed in vivo signal data is going to be a necessity.

With regards to sensory feedback in bioelectronics, in which the system input is an electronic sensor signal and the output is a pattern of stimulation to be delivered to a nerve, better bioelectrical models alone may not solve all of the problems in modern interfaces (Svensson et al., 2017). There are a select few human amputees who have received implantable somatosensory neurostimulators that are connected to prosthetic limbs, either chronically with devices like the e-OPRA or temporarily with experimental devices. Stimulating the peripheral nerves at different locations with various intensities has been shown to elicit sensations in the user that feel as if they are coming from the phantom limb (Iskarous & Thakor, 2019). Users have reported that the feedback from their prostheses sometimes feels like naturalistic touch or pressure, but it can also feel “electrical” or “buzzing” (Mastinu et al., 2020). Despite this synesthesia, the artificial restoration of sensation has repeatedly been shown to improve the utility and the embodiment of prosthetic limbs (Petrini et al., 2019). When it comes to the engineering of sensory feedback, there are two separate-yet-similar tracks one can go down: somatosensory perception or proprioception.

Perception is primarily associated with the previously-mentioned issue of stimulatory pulses feeling “tingly” when described by test subjects. Normal sensory neurons operate independently, but stimulating electrodes all have some current leak that can impact multiple nearby cells. This leads to sensory feedback that is intended to represent *indenting* pressure but instead causes the perception of a *pulsating* pressure or some other synesthesia that may not be accurately captured in words. This issue could be addressed by better microfabrication of more selective electrodes or by changing the stimulation mode entirely. Some research in the cochlear implant space has investigated the “steering” of stimulation currents in order to achieve higher precision (Berenstein et al., 2008). Safe direct current stimulation has also been explored as a possible method for selectively exciting *and* inhibiting neurons (Fridman & Della Santina, 2013; Aplin & Fridman, 2019). It is not yet known how these different types of stimulation might affect somatosensory perception.

As for proprioception, this is the sensory feedback that in normal tissue provides information to the CNS about the limb’s position in space. Sensory information about different muscle lengths and tendon stretch is subconsciously processed so that we can feel our limbs as a complete part of us even when we cannot see or directly feel them. So far there has only been one good solution to introducing artificial proprioception into artificial limbs, which is the agonist-antagonist myoneural interface (AMI) from the Herr group at the Massachusetts Institute of Technology (Martinez-Villalpando & Herr, 2009). The AMI is an interface that is constructed during below-the-knee amputation surgeries. Nearby muscles are surgically reconstructed so that they contract in opposition when a prosthetic foot is commanded to flex or extend such that the muscle contractions closely mimic the proprioception of foot dorsiflexion and extension (Srinivasan et al., 2020). This is an inspiring technique, but the wide variety in anatomy among both upper- and lower-limb amputees makes it so that the AMI may not easily be translated to all types of prosthetic limbs.

## Conclusion

The AMI is an appropriate segue into the concluding statements of this discussion because it is one of many examples demonstrating how the surgical reconstruction of neuromuscular tissue plays a large role in the future of bioelectronic interfaces. This idea circles back to the beginning of our article in which we point out that most of the biological tissue being targeted by bioelectronic devices has had to undergo some form of regeneration. Therefore, it seems imperative that biomedical engineers should consider both artificial electronics as well as living tissue to be within their BEM toolbox. Fortunately, there is a growing trend of biomedical engineers having the opportunity to work closely with reconstructive surgeons to solve problems in BEM (and some of the fruits from such collaborations have certainly been highlighted herein) (Vu et al., 2020; Ortiz-Catalan et al., 2020; Feussner et al., 2019). Medical universities have now become good places to partner the two professions so that they may work in tandem to find better solutions than ever before for nerve damage and amputation. This trend is a step in the right direction for the evolution of bioelectronics because these partnerships represent the best examples of the incredible healing processes innate to biological systems being leveraged to improve the body’s ability to interface with life-altering technology.

## Data Availability

Not applicable.

## Abbreviations

BEM: Bioelectronic medicine
TMR: Targeted muscle reinnervation
NMJ: Neuromuscular junction
RPNI: Regenerative peripheral nerve interface
VDMT: Vascularized denervated muscle target
MU: Motor unit
ATP: Adenosine triphosphate
EMG: Electromyography
PNS: Peripheral nervous system
CNS: Central nervous system
ALS: Amyotrophic lateral sclerosis (a.k.a. “Lou Gehrig’s disease”)
ENG: Electroneurography
AP: Action potential
STEER: Substrate targeted electrode reinnervation
sEMG: Surface EMG
MUAP: Motor unit action potential
eEMG: Epimysial EMG
SNR: Signal to noise ratio
iEMG: Intramuscular EMG
AI: Artificial intelligence
MRI: Magnetic resonance imaging
AMI: Agonist-antagonist myoneural interface
